# Implantable cardioverter defibrillators at the end of life: future perspectives on clinical practice

**DOI:** 10.1007/s12471-020-01438-6

**Published:** 2020-06-16

**Authors:** R. Stoevelaar, A. Brinkman-Stoppelenburg, R. L. van Bruchem-Visser, A. G. van Driel, R. E. Bhagwandien, D. A. M. J. Theuns, J. A. C. Rietjens, A. van der Heide

**Affiliations:** 1 Department of Public Health, Erasmus MC, Erasmus University Medical Centre, Rotterdam, The Netherlands; 2 Department of Internal Medicine, Erasmus MC, Erasmus University Medical Centre, Rotterdam, The Netherlands; 3 Department of Cardiology, Albert Schweitzer Hospital, Dordrecht, The Netherlands; 4 Rotterdam University of Applied Sciences, Rotterdam, The Netherlands; 5 Department of Cardiology, Erasmus MC, Erasmus University Medical Centre, Rotterdam, The Netherlands

**Keywords:** Implantable cardioverter defibrillator, Decision-making, Withholding treatment, Palliative care, Advance care planning, End-of-life care

## Abstract

The implantable cardioverter defibrillator (ICD) is effective in terminating life-threatening arrhythmias. However, in the last phase of life, ICD shocks may no longer be appropriate. Guidelines recommend timely discussion with the patient regarding deactivation of the shock function of the ICD. However, research shows that such conversations are scarce, and some patients experience avoidable and distressful shocks in the final days of life. Barriers such as physicians’ lack of time, difficulties in finding the right time to discuss ICD deactivation, patients’ reluctance to discuss the topic, and the fragmentation of care, which obscures responsibilities, prevent healthcare professionals from discussing this topic with the patient. In this point-of-view article, we argue that healthcare professionals who are involved in the care for ICD patients should be better educated on how to communicate with patients about ICD deactivation and the end of life. Optimal communication is needed to reduce the number of patients experiencing inappropriate and painful shocks in the terminal stage of their lives.

## The implantable cardioverter defibrillator at the end of life

The implantable cardioverter defibrillator (ICD) is highly effective in terminating potential life-threatening arrhythmias. However, for patients at the end of life, the goal of the ICD—prolonging survival—may no longer be appropriate. Guidelines recommend early and regular discussion of the appropriateness of the ICD shock function throughout the disease trajectory and subsequently deactivation of the ICD at the end of life [1, 2]. The Dutch Association of Cardiology (NVVC) released a guideline in 2013 [3] discussing the implications of the ICD at the end of life as well as the indication for and consequences of deactivating the device, but above all stresses the importance of discussing deactivation. Such discussions could be initiated before implantation (supported by a written patient folder), at follow-up (e.g. changing health, ICD battery change), and when entering the palliative and terminal phase. A recent Dutch study showed that only 35% of deceased ICD patients had discussed ICD deactivation. Further, 42% of patients had their ICD deactivated, and 9% experienced shocks in their last month of life [4], which have been reported as distressing for both patients and relatives [5]. In this point-of-view article, we discuss the barriers experienced by Dutch healthcare professionals to discussing ICD deactivation and provide future perspectives on clinical practice.

## Difficulties in discussing ICD deactivation

It is important that discussions on ICD deactivation are conducted before the actual deactivation [4]. However, healthcare professionals struggle with such discussions, while many patients indicate that they want to be informed about this topic [6]. Some research has described the potential barriers healthcare professionals experience to discussing ICD deactivation [7–9]. These include having too little experience or knowledge with regard to talking about deactivation [10–16], not feeling comfortable discussing the topic [14, 17–20], a predominant focus on cure and on the benefits of ICD therapy [10, 12, 18, 21, 22], having too little time to hold this conversation [12, 18, 19, 21], uncertainty about predicting patients’ disease trajectory [10, 12, 13, 21], being afraid of taking away hope [10, 12, 13, 19, 21], not knowing at what stage this conversation is appropriate [10], not knowing who is responsible for these conversations [10, 15], a lack of multidisciplinary cooperation [18], and a stressful work environment and high workload [23]. However, almost all of these studies were conducted in the USA, and no research has yet been performed in the Netherlands. Given the open culture in the Netherlands with regard to end-of-life decision-making [24], it is remarkable to see that ICD deactivation discussions do not occur often.

### Barriers hindering Dutch healthcare professionals from discussing ICD deactivation

We examined the experiences of Dutch healthcare professionals with ICD deactivation discussions in focus group meetings and individual interviews. Healthcare professionals were recruited via e‑mail in an academic and non-academic hospital and subsequently by using a snowball approach. Participants had to have experience in care for ICD patients at the end of life. Eighteen healthcare professionals participated in individual interviews, and two focus group meetings were held with four participants each. Interviews and focus groups were conducted between October 2017 and January 2018 using a predefined semi-structured topic list. Focus groups were led by experienced moderators (A.v.d.H. and J.A.C.R.), and the interviews were conducted by a psychologist (R.S.). Data were analysed using the constant comparative method. The characteristics of the participating healthcare professionals can be found in Tab. 1.

**Table 1:** Characteristics of participating healthcare professionals (*n* = 26)

Male gender	14 (54%)
Age (mean, SD)	47.0 (11.3)
Years of experience (mean, SD)	14.1 (8.5)
*Profession*	
– Physician^a^	11 (42%)
– Nurse^b^	11 (42%)
– ICD technician	4 (15%)
*Workplace*	
– Non-academic hospital	12 (46%)
– Academic hospital	8 (31%)
– Hospice	3 (12%)
– General practitioner office	2 (8%)
– Care home	1 (4%)

*ICD* implantable cardioverter defibrillator

^a^4 cardiologists, 4 elderly care physicians, 2 general practitioners, 1 oncologist

^b^5 cardiology nurses, 4 nurse specialists, 2 ICD nurses

All healthcare professionals reported experiencing some barriers to discussing ICD deactivation. Perceived barriers were related to clinical practice, the patient, or societal factors (Tab. 2). A frequently reported barrier relating to clinical practice was experiencing a lack of time to discuss this topic. A nurse specialist in palliative care observed: ‘*It calls for different planning of consultation hours, because those conversations take more time, and the consultation hours are not really designed for that*’. Other healthcare professionals described having a lack of knowledge about the ICD and the end of life, such as two elderly care physicians, who indicated that they were ‘unconsciously incompetent’. A cardiologist pointed out that it is difficult to find the right moment to discuss ICD deactivation, and that it might be inappropriate to discuss this topic during routine appointments. However, the pre-implantation conversation held with all patients was also regarded as inappropriate by some, who stated that this conversation should focus on practical aspects of the ICD. Another barrier reported was the fragmentation of healthcare and the predominant focus of healthcare professionals on their own discipline, which sometimes leads to overlooking the patient as a person. A nurse specialist remarked: ‘*The cardiologist only looks at the heart, the pulmonologist only looks at the lungs … everybody looks at his or her own small piece … we need to look at the patient as a whole. And that is sometimes not done*’. Other barriers related to clinical practice were concerns about taking away patients’ hope, difficulties in predicting the disease trajectory, a lack of experience in conducting such conversations, a lack of (awareness of) guidelines (only nine healthcare professionals mentioned the guideline of the NVVC), protocols which focus only on medical issues, and frequent staff turnover.

**Table 2:** Barriers for healthcare professionals to discussing deactivation of the implantable cardioverter defibrillator (*ICD*)

*Clinical practice*
Lack of time
Lack of knowledge about ICD in last phase of life
Difficulty finding the right moment to discuss deactivation
Lack of communication/coordination between healthcare professionals
Little insight into what other healthcare professionals do
Focus on practical matters
Focus on own discipline (fragmentation)
Being afraid to take away patients’ hope
Focus of cardiology on saving lives
Difficulty predicting patients’ disease trajectory
Feeling uncomfortable discussing last phase of life
Lack of experience discussing last phase of life
Focus on life-saving potential of ICD
Too little education on last phase of life, palliative care, and communication
Lack of (awareness of) guidelines
Uncertainty about who is responsible
Poor relationship with the patient
Lack of facility for a calm conversation
Difficulty stopping treatments
Protocols focus on medical aspects
Too much staff turnover
*Patients*
Reluctance to discuss/think about topic
Last phase of life not yet relevant/focused on practical matters
Overestimating life-saving character of ICD
Lack of knowledge about deactivation
Young age of patient
Topic too emotional
Culture/religion
Association with euthanasia
Lack of knowledge about what is medically possible
*Society*
Medicine/society focused too much on treatment/cure

Healthcare professionals also experienced barriers related to the patient: they felt that patients are reluctant to discuss ICD deactivation and the end of life. Some argued that patients tend to overestimate the life-saving ability of the ICD and think that, if the ICD is deactivated, they will immediately die. Also, many patients do not seem to think the topic is relevant yet. At the start of the treatment, patients are focused on living and the practical implications of the ICD. Later on in the disease trajectory, however, many patients are still not thinking about the end of life. A cardiologist compared this with retirement income: ‘*There are certain things, and the same goes for retirement income, we know it is important, but did you ever delve into how much you will actually receive?. … It is very difficult to motivate yourself to think about that in depth*’.

Attitudes in society towards death and dying were also mentioned as barriers to discussing ICD deactivation. Several clinicians indicated that medicine and society are predominantly focused on treating and curing illness, and length of life is often viewed as being more important than quality of life. An oncologist declared: ‘*Death should be a much more integral topic during life. We all want to be young forever, have no wrinkles, and whatever. … We want to overcome everything, overcome cancer, get cancer out of the world … it is nonsense. … We get cancer. It is part of our lives*’. A nurse specialist in palliative care said: ‘*We can do everything, but not everything we can do is always appropriate. When you are 92, do you have to have a new aortic valve or a new ICD? And another, and another? How realistic is that?*’. It was pointed out that no one can live forever, and that we need a different approach towards death and dying.

### Future perspectives

Despite guidelines on how to adequately address deactivation of the ICD shock function at the end of life, many patients never discuss ICD deactivation and die with an active ICD, some after experiencing painful shocks [4–6]. Palliative care, the end of life and advance care planning [25] are atypical subjects in the highly technological field of cardiology, but are of great importance [26]. However, healthcare professionals experience barriers to discussing these topics. Action is needed to increase attention to these topics and to overcome barriers. Educating healthcare professionals about the importance of discussing ICD deactivation and the last phase of life is needed, and can increase their involvement in advance care planning [27, 28].

A recent UK study by Javaid and colleagues reported on an easy-to-implement but effective programme to improve the attention given to ICD deactivation [29]. This programme encompassed: (1) raising awareness and increasing knowledge about ICD deactivation by presenting research and guidelines to different medical departments; (2) e-mailing all staff who were not able to be present during these presentations; (3) developing and distributing informative posters about the ICD and end-of-life care on medical wards; and (4) offering teaching and checklists to staff working on medical wards. After implementation of this programme, ICD deactivation increased from 0 to 54%, and none of the 13 patients studied experienced painful shocks during the last month of their lives. Although this study was small, the results are promising. Healthcare professionals in the Netherlands taking care of ICD patients should critically review how care at the end of these patients’ lives is organised. To further facilitate and initiate advance care planning discussions, consultation schedules and the role of nurses might be revisited, since they might be well suited to initiate discussions with patients regarding the future role of their ICD [18, 21, 30]. Advance care planning and discussions about end-of-life care should become an integral part of cardiological care. Only then can we reduce the number of patients experiencing inappropriate and painful shocks at the end of their lives.
